# The effect of restrictive vs. liberal fluid protocols on ocular parameters in patients undergoing prone spine surgery: a randomized controlled trial

**DOI:** 10.1186/s13741-023-00310-6

**Published:** 2023-06-12

**Authors:** Xiao-Yu Yang, Miao-Miao Wei, Hong Tan, Hai-Lian Wang, Meng-Qiang Luo, Ming Xu, Ying-Wei Wang

**Affiliations:** Department of, Anaesthesiology, Huashan Hospital Fudan University, 12 Middle Wulumuqi Road, Shanghai, 200040 China

**Keywords:** Fluid therapy, Intraocular pressure, Optic nerve, Postoperative complications, Prone position

## Abstract

**Background:**

Elevated intraocular pressure (IOP) and optic nerve edema occurring during prone surgeries may cause ocular and optic nerve ischaemia injury. We hypothesized that a liberal fluid protocol might further increase IOP and optic nerve sheath diameter (ONSD) than a restrictive fluid protocol for patients in the prone position.

**Methods:**

A single-centre, prospective and randomized trial was conducted. Patients were randomly allocated into 2 groups: the liberal fluid infusion group, in which repeated bolus doses of Ringer’s lactate solution were given to maintain pulse pressure variation (PPV) within 6~9%, and the restrictive fluid infusion group, where PPV was maintained within 13–16%. IOP and ONSD were measured in both eyes at 10min after the anaesthesia induction in the supine position, 10min after the prone position placement, and 1h and 2h since the prone position was placed, at the conclusion of surgery, and returned to the supine position.

**Results:**

A total of 97 patients were recruited and completed the study. IOP increased significantly from 12±3mmHg in the supine position to 31±5 mmHg (*p*<0.001) at the end of surgery in the liberal fluid infusion group and from 12±2 to 28±4 mmHg (*p*<0.001) in the restrictive fluid infusion group. There was a statistically significant difference in the change of IOP over time between the two groups (*p*=0.019). ONSD increased significantly from 5.3±0.3mm in the supine position to 5.5±0.3mm (*p*<0.001) at the end of surgery in both groups (both *p*<0.001). There was no statistically significant difference in the change of ONSD over time between the two groups (*p*>0.05).

**Conclusions:**

Compared to the restrictive fluid protocol, the liberal fluid protocol increased IOP but not ONSD in patients undergoing prone spine surgery.

**Trial registration:**

The study was registered in ClinicalTrials.gov (https://clinicaltrials.gov) prior to patient enrollment, ID: NCT03890510, on March 26, 2019. The principal investigator was Xiao-Yu Yang.

**Supplementary Information:**

The online version contains supplementary material available at 10.1186/s13741-023-00310-6.

## Introduction

Postoperative visual loss (POVL) is a rare but disabling complication (Lee 2013; Practice advisory for perioperative visual loss associated with spine surgery 2019). The highest incidence of POVL occurs after spine and cardiac surgeries (Shen et al. 2009). The estimated rate of POVL with several types of spine surgery is as high as 0.2% (Patil et al. 1976; Li et al. 2015). Potential preventive interventions (Practice advisory for perioperative visual loss associated with spine surgery 2019) and head-positioning devices (Uribe et al. 2012) have been recommended and utilized in clinical practice, but POVL still occurs unpredictably after non-ophthalmologic surgeries (Li et al. 2015; Nuzzi and Tridico 2016).

Ischaemic optic neuropathy (ION) is the most frequent cause of POVL that associated with spine surgery (Rubin et al. 2016; Nandyala et al. 2014; Goyal et al. 2019). Anterior ION typically has optic disc edema upon symptom onset (Roth and Moss 2018; Nickels et al. 2014). While in posterior ION, optic disc swelling is absent on ophthalmoscopic examination and MRI describes nerve enlargement or perineural enhancement suggesting edema in some patients (Roth and Moss 2018; Kamel and Barnette 2014). The most important risk factors of POVL are the prone position and steep Trendelenburg position (Lee 2013; Li et al. 2015; Nuzzi and Tridico 2016; Lee et al. 2006). The etiology behind ION remains uncertain whereas seeming to be multifactorial (Lee et al. 2006; Roth 2009). Most patients suffered from bilateral rather than unilateral ION after prone spine surgery and were relatively healthy, suggesting that intraoperative systemic pathophysiologic changes and/or individual anatomic variations may have a greater impact than preexisting comorbidities in developing ION (Kamel and Barnette 2014; Lee et al. 2006; Cheng et al. 2001; American Society of Anesthesiologists Task Force on Perioperative Visual Loss 2012).

Massive fluid administration is common in patients with ION after prone spine surgery, indicating that intraoperative fluid replacement may have an effect on POVL (Lee 2013; Roth and Moss 2018). Clinical researches reveal that large amounts of crystalloid infusion can increase intraocular pressure (IOP) and periorbital edema in patients undergoing cardiopulmonary bypass, which may cause ocular and optic nerve ischaemia injury (Practice advisory for perioperative visual loss associated with spine surgery 2019). However, whether a liberal fluid protocol with crystalloids in clinically acceptable range can induce an increase in IOP and optic nerve edema is not clear in anaesthetized patients in the prone position. Therefore, we hypothesize that a liberal fluid protocol could further increase IOP and optic nerve sheath diameter (ONSD) than a restrictive fluid protocol in patients under anaesthesia in the prone position.

## Materials and methods

### Study design

This single-centre, prospective and randomized trial was reviewed and approved by the institutional ethics committee of Huashan Hospital, Fudan University (KY2018-333). After written informed consents were obtained, patients were enrolled in the study. The inclusion criteria were aged from 18 to 60 years, female or male, American Society of Anaesthesia (ASA) physical status classes I or II, and scheduled for elective lumbar spine surgery in the prone position under general anaesthesia. Exclusion criteria were preexisting eye disease except for myopia under 500 or previous eye surgery; pregnancy or breastfeeding; known allergy to latex or Ringer's lactate solution; hyperlacticaemia, uncontrolled chronic diseases (such as hypertension, diabetes mellitus, arrhythmia, cardiovascular disease and chronic pulmonary disease), swelling of any body part, abnormal of liver or renal function, and anaemia; body mass index (BMI) >30; expected operation time >6 h; estimated intraoperative blood loss >1000ml; and taking part in other clinical trials in the last 3 months or at present. If the recruited patients received a blood transfusion or their hematocrit level dropped under 0.3 during surgery; their baseline IOP or ONSD were abnormal or different between two eyes (IOP difference >3mmHg or ONSD difference >0.5mm between right and left eyes); or their surgery time was less than 2 h or more than 6 h, they would be excluded from the study. The study was registered in ClinicalTrials.gov (https://clinicaltrials.gov) prior to patient enrollment, ID: NCT03890510. This manuscript adheres to the applicable CONSORT guidelines.

The patients were randomly allocated with a 1:1 ratio into two parallel intervention groups: liberal fluid infusion group (target value of pulse pressure variation, PPV: 6–9%) and restrictive fluid infusion group (target value of PPV: 13–16%), using a computer-generated random number table at the day of surgery when arriving at the operation room. The investigator who enrolled participants and assigned participants to interventions did not perform anaesthesia to participants or measure outcomes values.

Standardized anaesthesia in accordance with institutional protocols was performed to all patients. In brief, patients were fasted for at least 8 h before anaesthesia. A 20-G radial artery catheter was placed under local anaesthesia and connected to a pressure sensor kit (TransStar, Smiths Medical ASD, Inc. Dublin, USA) and a monitoring system (B650, General Electric Company, Boston, USA) to obtain arterial blood pressure and PPV continuously. General anaesthesia was induced with intravenous midazolam of 0.05mg•kg^−1^, propofol of 2–3 mg•kg^−1^, fentanyl of 3μg•kg^−1^ and rocuronium of 0.6mg•kg^−1^. After endotracheal intubation, anaesthesia was continued with sevoflurane (end-tidal concentration titrated between 0.5 and 1.5 minimal alveolar concentration) and oxygen (fractional inspired O_2_=0.6) to maintain bispectral Index (BIS) between 40~50. Patients were mechanically ventilated. Volume-guaranteed pressure control ventilation was used, and tidal volume was set at 8ml•kg^-1^. PEEP was not used in any patients. The respiratory rate was set to 10 times per minute at the beginning and adjusted afterward to maintain normocapnia (end-tidal CO_2_, EtCO_2_, in the range of 30–35 mmHg). Airway peak pressure was controlled lower than 25 cmH_2_O by adjusting inspire/expire time ratio. After the induction of anaesthesia, patients were turned prone appropriately on a Jackson table. In order to prevent extraocular pressure, their heads were held by a square-shaped gel headrest. Their eyes were accessible from beneath during the procedures. The heads were placed at the level of the heart in a neutral position without neck flexion or extension. The position of the head and eyes was checked every 30 min during the whole prone procedure. Additional doses of fentanyl and rocuronium were titrated to maintain an adequate level of analgesia and muscle relaxation (the train-of-four, TOF target: 1). Body temperature was maintained between 36 and 37℃ with a warm air blanket device. Electrocardiogram, pulse oximetry, capnography, body temperature, radial arterial blood pressure and PPV were monitored continuously during anaesthesia. Blood pressure was maintained within ±20% of the awake value and systolic blood pressure above 90 mmHg. Deliberate hypotension was avoided in all patients. Intraoperative hypotension was corrected with multiple doses of phenylephrine and/or ephedrine. Patients in both groups received Ringer’s lactate solution continuously with a basal infusion speed at 1ml•kg^-1^•h^-1^. Repeated bolus doses of 250ml Ringer’s lactate solution were given to maintain PPV within 6~9% in the liberal fluid infusion group and PPV within 13–16% in the restrictive fluid infusion group. Haemoglobin, hematocrit values and blood gas were monitored at an hour interval during surgery. All lumbar spine surgeries were conducted by one group of experienced orthopedists.

### Data collection

Characteristics of the patients were collected from medical records and preoperative interviews by investigators. Intraoperative parameters were recorded by the anaesthesiologist in charge of the anaesthesia. IOP and ONSD were measured by an experienced investigator who was blinded to the fluid management and did not involve in the anaesthesia. IOP was measured with a handheld tonometer (Tono-pen AVIA, Reichert Inc, NY, USA) which could be used in any position. The tonometer was calibrated according to the manufacturer’s manual before each application. ONSD was measured with a high-frequency probe of ultrasound equipment (Sonosite EDGE, Fujifilm Inc., WA, USA). During ONSD measurement, closed eyelids were covered with a thin plastic cover to separate ultrasound gel from the eyes, and pressure on the eye globes was avoided. After locating the widest ONS cross-section, the ONSD was measured 3 mm behind the lamina cribrosa by calculating the distance between the hypoechogenic borders of the ONS. A mean of 3 repeated measurements was recorded. IOP and ONSD were measured in both eyes at 6 time points: 10min after anaesthesia induction in the supine position; 10min after the prone position placement; 1h and 2h since the prone position was placed, at conclusion of surgery; and 10min after returning to the supine position. At each time point, mean artery pressure (MAP), heart rate (HR), pulse oximetry, EtCO_2_ and peak inspiratory pressure were also recorded. Time-weighted average (TWA) values of the collected data for each patient were calculated as follows: TWA = (t1X1 + t2X2 + … tnXn)/(t1 + t2 + …+ tn), where Xn is the value of the interested variable during the *n*th interval and tn is the duration of the *n*th interval. The time interval was 5 min. Estimated blood loss, baseline amount of fluid administration, number of fluid boluses and the overall amount of boluses, total dose of vasopressor use and urine volume were recorded at the end of surgery. Patients were asked for eye discomfort or vision changes and checked for possible eye complications at the postoperative care unit after emergence and in the ward the next day.

The primary outcome of the study is the change of IOP over time between groups in the prone position. The secondary outcome of the study is the change of ONSD over time between groups in the prone position.

### Power calculation

According to a previous study of prone spine surgery under general anaesthesia, the average IOP in the prone position was 35.2±8mmHg (Farag et al. 2012). We assumed that the 5mmHg difference of IOP between groups (33mmHg vs. 38mmHg expected) in the prone position was of clinical significance. Power analysis calculation suggested that at least 42 patients should be recruited per group to provide a power of 80% (*β*=0.2), with a two-sided confidence interval of 95% (*α*=0.05). We aimed to include at least 94 patients in the trial to allow a margin of ‘statistical safety’ and compensate for dropouts.

### Statistical analysis

Descriptive statistics were presented as mean ± SD (standard deviation) for continuous data and number (proportion) for categorical data. The statistical analysis was performed using Stata 15.1 (StataCorp LLC Texas, USA). Normality was tested by the Kolmogorov–Smirnov analysis. Comparisons of continuous data between groups were examined using *t* test or Wilcoxon rank sum test and post hoc Bonferroni correction. A *χ*^2^ analysis or Fisher’s exact test was used to analyse categorical data between groups. IOP and ONSD of the right and left eyes were compared with paired *t* test or Wilcoxon sign rank test with Bonferroni correction. Linear mixed effects models were used to compare outcome variables that measured repeatedly across successive time points (IOP, ONSD, MAP, HR, EtCO_2_, PaCO_2_, haemoglobin, hematocrit and peak inspiratory pressure). A *p* value <0.05 was considered statistically significant.

## Results

**Fig. 1:**
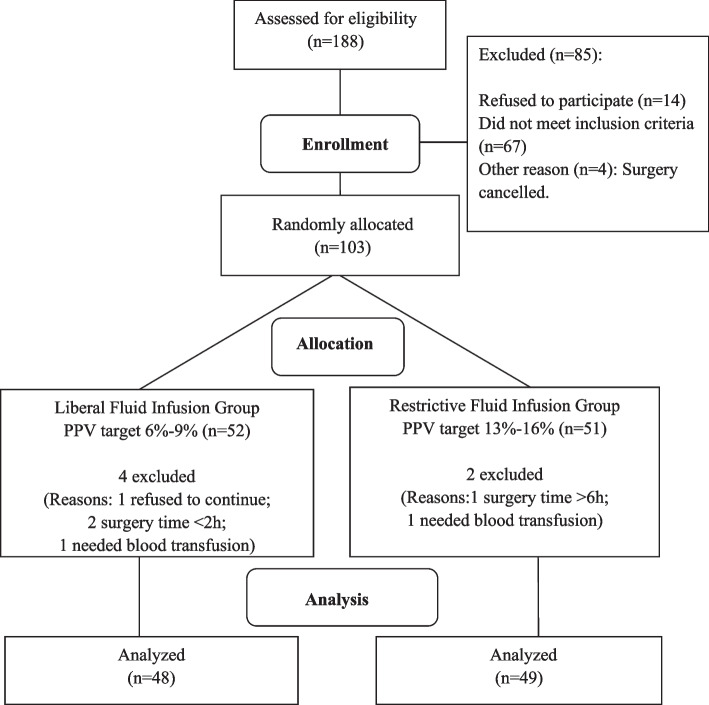
Study workflow

A total of 97 patients were recruited and completed the study in Huashan Hospital from May 2019 to August 2020 (Fig. 1). Patient characteristics are shown in Table 1. The baseline characteristics were comparable between the two groups. The flow chart of the study is shown in Fig. 1.

**Table 1:** Patient characteristics

	Liberal fluid infusion group (PPV target 6–9%) *n*=48	Restrictive fluid infusion group (PPV target 13–16%) *n*=49
Age (year)	51±8	49±10
Gender (*n*)		
Male	21 (0.44)	25 (0.51)
Female	27 (0.56)	24 (0.49)
Height (cm)	166.4±9.2	167.5±9.5
Weight (kg)	65.7±11.0	67.7±10.9
Body mass index (BMI) (kg•m^-2^)	23.6±2.5	24.0±2.4
ASA physical status (*n*)		
I	26 (0.54)	29 (0.59)
II	22 (0.46)	20 (0.41)
Comorbidity (*n*), total	22 (0.46)	20 (0.41)
Hypertension	18 (0.38)	16 (0.33)
Diabetes mellitus	6 (0.13)	9 (0.18)
Asthma	2 (0.04)	0 (0)
Number of lumbar segments (*n*)		
1	29 (0.60)	36 (0.73)
2	17 (0.35)	11 (0.22)
3	2 (0.04)	2 (0.04)

Data are presented as mean±standard deviation or *n* (proportion)

Intraoperative profiles are presented in Table 2. The groups were similar in terms of most surgery- and anaesthesia-related profiles. Because of different fluid infusion protocol, the number of fluid boluses (*p*<0.001), total bolus volume (*p*<0.001), urine output (*p*<0.001) and blood haematocrit (*p*=0.008) over time were significantly different between the two groups.

**Table 2:** Intraoperative profiles

	Liberal fluid infusion group (PPV target 6–9%) (*n*=48)	Restrictive fluid infusion group (PPV target 13–16%) (*n*=49)
Anaesthesia time (min)	181±29	178±23
Operation time (min)	139±27	135±22
Prone time (min)	154±28	149±23
End-tidal sevoflurane concentration (%)	2.0±0.3	1.9±0.4
Fluid infusion		
Baseline (ml)	211±40	203±38
Number of boluses (n)	7±2	5±2^^^^^
Total bolus volume (ml)	1747±517	1099±395^^^^^
Blood loss (ml)	150±67	162±70
Urine output (ml)	302±163	163±63 ^^^^^
Body temperature (°C)	36.5±0.2	36.5±0.3
PPV (%)		
Baseline	14±2	15±2
Intraoperative time-weighted average	8±1^***^	14±1^^^^^
Haematocrit (%)		
Baseline before anaesthesia	39.9±1.1	40.2±1.6
During anaesthesia		
1 h	36.2±1.0^***^	37.1±1.2^***^^
2 h	35.6±1.1^***^	36.9±1.1^***^^^
End of surgery	35.4±1.3^***^	36.8±1.1^***^^^
PaO_2_ (mmHg)		
Baseline before anaesthesia	85.1±9.7	85.9±11.2
During anaesthesia		
1 h	297.1±63.4^***^	297.6±55.9^***^
2 h	294.4±61.9^***^	300.8±49.8^***^
End of surgery	290.5±60.2^***^	292.8±46.4^***^
PaCO_2_ (mmHg)		
Baseline before anaesthesia	36.3±2.4	36.1±2.4
During anaesthesia		
1 h	36.9±2.4	37.3±1.5^**^
2 h	37.0±2.0	36.6±1.1
End of surgery	37.2±1.3 ^**^	36.3±1.4
Lactate (mmol•L^–1^)		
Baseline before anaesthesia	0.9±0.2	0.8±0.3
During anaesthesia		
1 h	1.2±0.2^***^	1.1±0.3^***^
2 h	1.2±0.2^***^	1.1±0.3^***^
End of surgery	1.2±0.2^***^	1.1±0.3^***^
Patients needed vasopressors (*n*)	39	40
Total ephedrine dose (mg)	3±4	3±3
Total phenylephrine dose (μg)	29±36	36±39

Data are presented as mean±standard deviation or *n* (proportion)

*PPV* pulse pressure variation

^*^p<0.05 compared to baseline, **p<0.01 compared to baseline, ***p<0.001 compared to baseline

^*p*<0.05 compared to the liberal fluid infusion group, ^^*p*<0.01 compared to the liberal fluid infusion group, ^^^*p*<0.001 compared to the liberal fluid infusion group

IOP and ONSD values of the left and right eyes were similar at all time spots in both groups (Table 3). So we used the average IOP and ONSD of the left and right eyes for analysis. During the study of the first three recruited cases, we found that at the end of anaesthesia BIS and TOF could hardly be maintained without extra doses of anaesthetics and muscle relaxants, which were considered unsafe for patients. Therefore, we measured IOP and ONSD as soon as patients were positioned supine instead of 10 min later. And the values measured at this time spot were excluded from statistics of significance.

**Table 3:** IOP and ONSD values of the left and right eyes

	Supine baseline	Prone				Supine end of anaesthesia
		10min	1 h	2 h	End of surgery	
Liberal fluid infusion group						
IOP (left) (mmHg)	12±3	20±4	27±4	30±5	31±5	30±5
IOP (right) (mmHg)	13±3	20±4	27±4	30±6	31±5	29±5
ONSD (left) (mm)	5.3±0.3	5.3±0.3	5.5±0.3	5.5±0.3	5.6±0.3	5.4±0.3
ONSD (right) (mm)	5.3±0.3	5.3±0.3	5.5±0.3	5.5±0.3	5.6±0.3	5.4±0.3
Restrictive fluid infusion group						
IOP (left) (mmHg)	12±3	19±3	24±4	27±4	27±4	27±4
IOP (right) (mmHg)	12±2	19±3	25±4	27±4	28±3	27±4
ONSD (left) (mm)	5.3±0.3	5.4±0.4	5.5±0.4	5.5±0.3	5.5±0.3	5.5±0.3
ONSD (right) (mm)	5.3±0.4	5.3±0.4	5.5±0.3	5.5±0.3	5.5±0.3	5.5±0.3

Data are presented as mean±standard deviation

*IOP* Intraocular pressure, *ONSD* Optic nerve sheath diameter

**Fig. 2:**
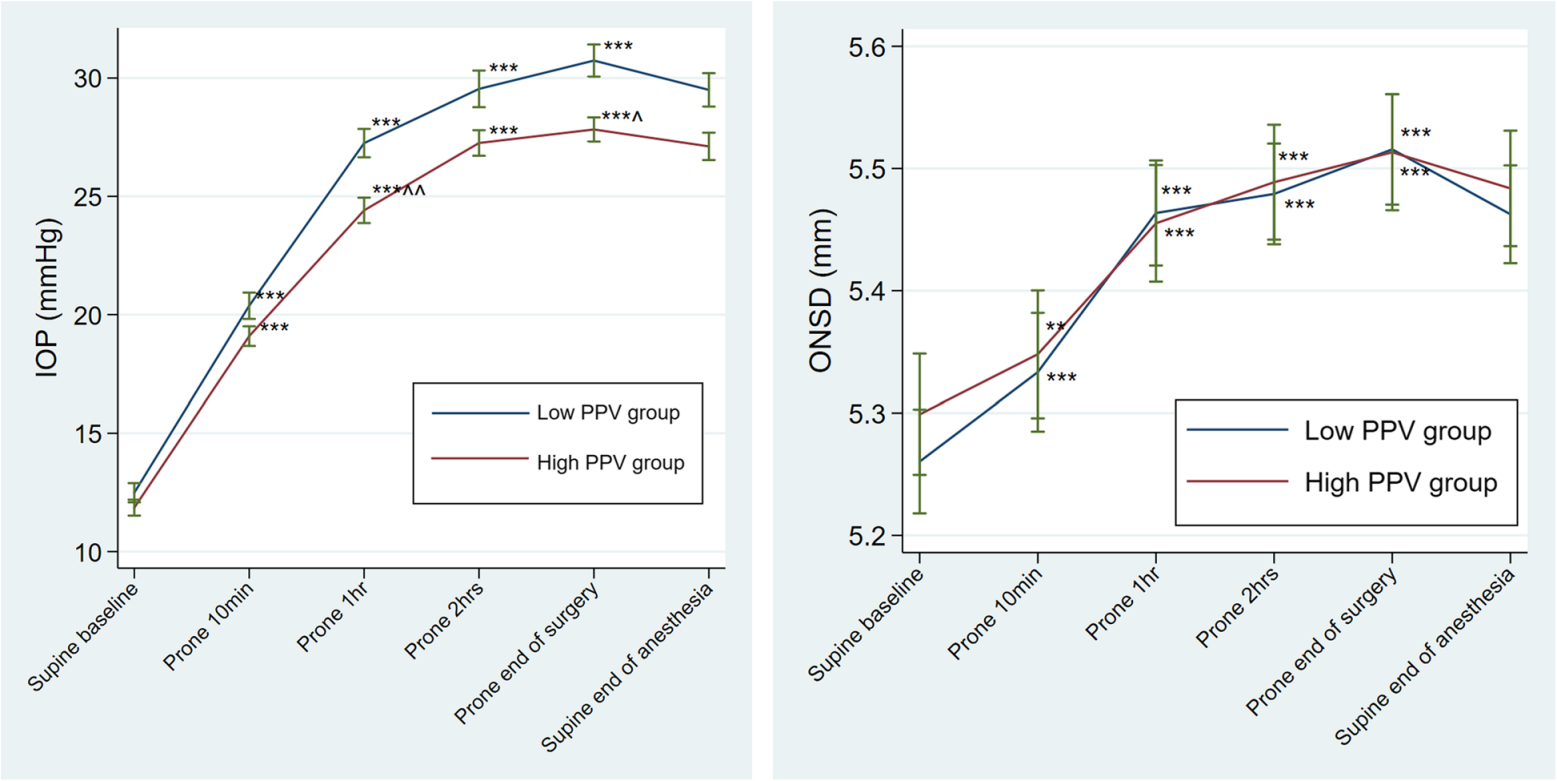
(1) IOP changes in the prone position of the low PPV group (liberal fluid infusion group) and the high PPV group (restrictive fluid infusion group). The significant difference over time between groups. (2) ONSD changes in the prone position of the low PPV group (liberal fluid infusion group) and high PPV group (restrictive fluid infusion group). No significant difference over time between groups. IOP intraocular pressure. PPV pulse pressure variation. ONSD optic nerve sheath diameter. **p<0.01 compared to baseline, ***p<0.001 compared to baseline; ^*p*<0.05 compared to the low PPV group, ^^*p*<0.01 compared to the low PPV group

IOP increased significantly from 12±3mmHg (liberal fluid infusion group) and 12±2mmHg (restrictive fluid infusion group) in the supine position to 31±5 mmHg (liberal fluid infusion group) and 28±4 mmHg (restrictive fluid infusion group) at the end of surgery in the prone position (both *p*<0.001 compared to baseline values) (Table A1). There was a statistically significant difference in the change of IOP over time between the liberal fluid infusion group and the restrictive fluid infusion group (*p*=0.019) (Fig. 2). ONSD also increased significantly with time in the prone position (from 5.3±0.3mm to 5.5±0.3mm at the end of surgery in both groups, *p*<0.001 (Table A1). However, ONSD only increased by 4% and there was no statistically significant difference in the change of ONSD over time between groups (*p*>0.05) (Fig. 2). Haemodynamic and ventilatory parameters were comparable between groups while patients were in the prone position (all *p*>0.05) (Table A1).

No ocular complications, including postoperative visual loss, or eye discomfort were observed or reported in the recovery room and during follow-up on the next day after surgery.

## Discussion

Our study demonstrated that liberal fluid administration with a PPV target of 6–9% can further increase IOP but not ONSD compared to restrictive fluid administration with a PPV target of 13–16% in patients undergoing spine surgery in the prone position.

IOP and ONSD have been shown to rise above the normal range with a time-dependent pattern in patients in the prone position (Cheng et al. 2001; Grant et al. 2010; Yoshimura et al. 2015). The prone position and the supine head-down (Trendelenberg) position are both risk factors of ION (Nuzzi and Tridico 2016; Blecha et al. 2017). Normal IOP ranges from 10 to 20 mmHg (Kamel and Barnette 2014). In anaesthetized patients, IOP can reach 40mmHg, almost doubled compared to the upper limit of the normal range, after 5 h in the prone position, although general anaesthesia has an obvious effect of lowering IOP (Cheng et al. 2001). A study observed that IOP, choroidal thickness and optic nerve diameter increased progressively with time in awake volunteers in the prone position (Grant et al. 2010). They reported an average of 36% increase in ONSD after 5 h in the prone position (Grant et al. 2010). Another study reported that the ONSD increased by 12.5% (0.6 mm) with Trendelenburg position (Kim et al. 2014).

Excessive volume of crystalloid infusion or intake may increase IOP and ONSD (Roth 2009; Brucculeri et al. 1999). In healthy volunteers, acute oral water loading (14 ml/kg) increases IOP (Roth 2009). On the contrary, exercise-induced dehydration can reduce IOP (Martin et al. 1999). A large multicentre case–control study reported that the percentage of crystalloid in the total fluid infusion volume was not statistically significant on developing ION, but higher colloid as a percentage of total nonblood infusion volume was associated with a reduced risk of developing ION (American Society of Anesthesiologists Task Force on Perioperative Visual Loss 2012). A randomized trial of patients undergoing complex prone spine surgery discovered no significant difference in intraoperative time-weighted average IOP but a lower increasing speed of IOP in the 5% albumin infusion group than the total crystalloid infusion group (Farag et al. 2012). Some researchers suggest to limit the volume of crystalloid solution to reduce the possibility of increased interstitial fluid and pressure in the orbit; (Weiskopf et al. 2007) however, no research has studied the effect of fluid volume on eye parameters during prone procedures.

In our study, IOP in both groups increased above the normal range after 1 h of the prone position and more than doubled at the end of surgery compared to the baseline value. The ocular perfusion pressure is estimated as the difference between MAP and IOP. An increase in IOP can decrease ocular perfusion pressure, which may contribute to retinal or anterior optic nerve ischaemia injury, especially in patients with existing ocular comorbidities or vascular risk factors (Nuzzi and Tridico 2016; Nandyala et al. 2014; Grant et al. 2010; Yoshimura et al. 2015). One of the possible reasons for the increase in IOP and ONSD in the prone position is the elevated venous pressure within choroid, sclera and optic nerve due to fluid retention (Nandyala et al. 2014; Lee et al. 2006; Cheng et al. 2001; Grant et al. 2010; Yoshimura et al. 2015). Baig et al. reported the possibility that fluid retention within the eyes and optic nerves induced by a prone position during surgery could be exacerbated by an overload of fluid replacement, thus increasing the risk of edema and compromising tissue oxygenation (Baig et al. 2007). Autoregulatory mechanisms of the optic nerve can normally offset slight perfusion deviations, but ischemic complications can occur due to external compression by edematous fluids or a watershed zone of optic nerve circulation (Lee 2013; Nickels et al. 2014; American Society of Anesthesiologists Task Force on Perioperative Visual Loss 2012; Grant et al. 2010). Some researchers have raised concern that prolonged elevation of intraocular venous pressure, increased interstitial fluid accumulation within the optic nerve and oedema of extraocular tissues in the enclosed bony optic canal may increase the risk of ION, although such speculation remains to be proven (Lee 2013; Nandyala et al. 2014; Goyal et al. 2019; Grant et al. 2010). We demonstrated that the increase of IOP over time was more severe in the liberal fluid infusion group than the restrictive fluid infusion group, indicating an effect of fluid volume on IOP. Based on our results, we suggest further research of fluid protocols for patients in the prone position with preexisting elevated IOP, such as glaucoma, high-grade hyperopia and retinopathy.

The increase of IOP that we observed was in accordance with those reported in other studies, whereas ONSD rose only 4% (0.2 mm) by average above the initial value in our study. The discrepancy may be the result of different study designs and patient populations. As far as we know, no other study has ever measured ONSD in the prone position during anaesthesia. The 36% increase of ONSD was observed in 10 awake volunteers at the end of 5 h prone position (Grant et al. 2010). Since general anaesthesia has a clear effect of lowering IOP, (Cheng et al. 2001) we cannot rule out the possibility that general anaesthesia also has an effect on ONSD. The 12.5% increase of ONSD during surgeries in the Trendelenburg position can be partly explained by elevated intracranial pressure due to the lower position of the head compared to the heart level, (Kim et al. 2014) while the head is at the same level of the heart in the prone position. In addition, elevated intrathoracic pressure and intraabdominal pressure may both increase intraocular venous pressure (Nandyala et al. 2014; Lee et al. 2006; Roth 2009). In our study, patients were positioned properly in the prone position, and the peak inspiratory pressure was controlled under 15 cm H_2_O on average. Therefore, it is reasonable that the increase of ONSD was much milder than those reported in previous papers. The clinical significance of such a slight increase of ONSD observed in our study may probably be minor.

PPV is a functional haemodynamic parameter based on arterial waveform analysis that can effectively evaluate fluid responsiveness (Chew and Åneman 2013; Perel et al. 2014). It is widely used in guiding goal-directed fluid therapy, which has been repeatedly proven to improve patient outcomes and reduce hospital stays in many types of surgical procedures (Perel et al. 2013). We used PPV targets in the study to divide patients into different fluid infusion volume groups and avoid severe fluid overload.

In the study, 6 patients were excluded after randomization according to our predefined protocols. The reasons were patient refusal, too short or too long surgery time, and requiring blood transfusion. We could not continue collecting their data after the exclusion, so an intention-to-treat analysis was not possible. In this case, our conclusion was drawn from a per-protocol analysis. Considering the post-randomisation exclusion rate was low and these patients did not have a uniform character, the possibility that the exclusions could bias the conclusion was low.

There are several limits of our study. First, although IOP was above the normal range in the prone position, the value at which IOP significantly influences the retinal blood flow and needs intervention is not certain. Ophthalmologists recommend to treat patients whose IOP>25mmHg during prone surgery (Nuzzi and Tridico 2016). Second, we did not measure IOP in the postoperative period; therefore, it was not clear how long the elevated IOP would last. Third, the difference of IOP changes between groups in our study was significant but not as much as we had expected, mainly because the average surgery time and accordingly the difference of fluid volume between groups were also lower than we formerly expected. If the time of staying in a prone position increased and the difference of fluid volume between groups widened, a greater IOP difference between groups could be assumed. Last, we did not find a significant difference in ONSD increase between different fluid infusion groups. However, the increase of ONSD might be significant in patients with BMI higher than 30 or with much lengthy prone procedures. Future studies may explore whether a similar intervention could lead to greater differences of IOP and ONSD changes in patients with more comorbidities or longer surgeries.

## Conclusion

Our study demonstrated that the liberal fluid protocol can further increase IOP but not ONSD compared to the restrictive fluid protocol in patients undergoing spine surgery in the prone position.

## Supplementary Information

**Additional file 1:** **Table A. 1.** IOP and ONSD outcomes and related measures. Description of data: A data table.

## Data Availability

The datasets used and/or analysed during the current study are available from the corresponding author on reasonable request.
