# Supplementary material for: The effect of restrictive vs. liberal fluid protocols on ocular parameters in patients undergoing prone spine surgery: a randomized controlled trial
Source: Perioper Med (Lond). 2023 Jun 12;12:23. doi: 10.1186/s13741-023-00310-6 (PMC10262396; doi:10.1186/s13741-023-00310-6)
Supplement: Supplementary file 1 — Additional file 1: Table A. 1. IOP and ONSD outcomes and related measures. Description of data: A data table. [file 13741_2023_310_MOESM1_ESM.docx]

Table A1. IOP and ONSD outcomes and related measures

|  | **Supine**  **Baseline** | | | **Prone** | | | | | | **Supine**  **End of anaesthesia** | |
| --- | --- | --- | --- | --- | --- | --- | --- | --- | --- | --- | --- |
|  |  |  |  | 10min | 1 hour | | 2 hours | | End of surgery |  |  |
| Mean IOP (mmHg) | |  |  | |  |  | |  | | |  |
| Liberal group | | 12±3 | 20±4*** | | 27±4*** | 30±5*** | | 31±5*** | | | 30±5 |
| Restrictive group | | 12±2 | 19±3*** | | 24±4***^^ | 27±4*** | | 28±4***^ | | | 27±4 |
| Mean difference  between groups | | 1(0, 2) | 1(-1, 2) | | 2(1, 4) | 2(0, 4) | | 2(1, 4) | | | 2(0, 4) |
| Mean ONSD (mm) | |  |  | |  |  | |  | | |  |
| Liberal group | | 5.3±0.3 | 5.3±0.3*** | | 5.5±0.3*** | 5.5±0.3*** | | 5.5±0.3*** | | | 5.5±0.3 |
| Restrictive group | | 5.3±0.3 | 5.3±0.4** | | 5.5±0.3*** | 5.5±0.3*** | | 5.5±0.3*** | | | 5.5±0.3 |
| Mean difference  between groups | | 0(-0.2, 0.1) | 0(0, 0.1) | | 0(0, 0.1) | 0(0, 0.1) | | 0(-0.1, 0.1) | | | 0(0, 0.1) |
| MAP (time-weighted average) (mmHg) | | |  | |  |  | |  | | |  |
| Liberal group | | 83±7 | 82±7 | | 81±8 | 82±5 | | 81±6 | | | 82±7 |
| Restrictive group | | 80±8 | 81±6 | | 82±7 | 82±7 | | 82±5 | | | 82±7 |
| Heart rate (beat per min) | |  |  | |  |  | |  | | |  |
| Liberal group | | 72±8 | 67±6** | | 66±5*** | 69±6 | | 70±6 | | | 69±5 |
| Restrictive group | | 69±8 | 67±8 | | 65±4** | 68±6 | | 68±5 | | | 68±4 |
| Pulse oximetry (%) | |  |  | |  |  | |  | | |  |
| Liberal group | | 100±0 | 100±1 | | 100±1 | 100±1 | | 100±0 | | | 100±0 |
| Restrictive group | | 100±0 | 100±0 | | 100±0 | 100±1 | | 100±0 | | | 100±0 |
| EtCO_2_ (mmHg) | |  |  | |  |  | |  | | |  |
| Liberal group | | 33±2 | 34±2 | | 33±2 | 34±1* | | 34±1*** | | | 33±2 |
| Restrictive group | | 33±2 | 34±2 | | 34±2 | 34±1** | | 34±1* | | | 34±1 |
| Peak inspiratory pressure (cm H_2_O) | | |  | |  |  | |  | | |  |
| Liberal group | | 14±2 | 14±1 | | 14±1 | 14±1 | | 14±1 | | | 14±1 |
| Restrictive group | | 15±2 | 15±2 | | 14±1 | 15±1 | | 15±1 | | | 14±1 |

Data are presented as mean±standard deviation or mean (95% confidence interval).

IOP: intraocular pressure. ONSD: optic nerve sheath diameter. MAP: mean artery pressure.

Liberal group: liberal fluid infusion group. Restrictive group: restrictive fluid infusion group.

*p<0.05 compare to baseline, **p<0.01 compare to baseline, ***p<0.001 compare to baseline;

^ p<0.05 compare to liberal fluid infusion group, ^^p<0.01 compare to liberal fluid infusion group.
